# Hydrogen sulfide: role in ion channel and transporter modulation in the eye

**DOI:** 10.3389/fphys.2012.00295

**Published:** 2012-07-25

**Authors:** Ya F. Njie-Mbye, Catherine A. Opere, Madhura Chitnis, Sunny E. Ohia

**Affiliations:** ^1^Department of Pharmaceutical Sciences, College of Pharmacy and Health Sciences, Texas Southern UniversityHouston, TX, USA; ^2^Department of Pharmacy Sciences, School of Pharmacy and Health Professions, Creighton UniversityOmaha, NE, USA

**Keywords:** cystine/glutamate antiporter, EAAT/GLAST transporter, hydrogen sulfide, ion channels, ocular tissues, cysteine transporter

## Abstract

Hydrogen sulfide (H_2_S), a colorless gas with a characteristic smell of rotten eggs, has been portrayed for decades as a toxic environmental pollutant. Since evidence of its basal production in mammalian tissues a decade ago, H_2_S has attracted substantial interest as a potential inorganic gaseous mediator with biological importance in cellular functions. Current research suggests that, next to its counterparts nitric oxide and carbon monoxide, H_2_S is an important multifunctional signaling molecule with pivotal regulatory roles in various physiological and pathophysiological processes as diverse as learning and memory, modulation of synaptic activities, cell survival, inflammation, and maintenance of vascular tone in the central nervous and cardiovascular systems. In contrast, there are few reports of a regulatory role of H_2_S in the eye. Accumulating reports on the pharmacological role of H_2_S in ocular tissues indicate the existence of a functional trans-sulfuration pathway and a potential physiological role for H_2_S as a gaseous neuromodulator in the eye. Thus, understanding the role of H_2_S in vision-related processes is imperative to our expanding knowledge of this molecule as a gaseous mediator in ocular tissues. This review aims to provide a comprehensive and current understanding of the potential role of H_2_S as a signaling molecule in the eye. This objective is achieved by discussing the involvement of H_2_S in the regulation of (1) ion channels such as calcium (L-type, T-type, and intracellular stores), potassium (K_ATP_ and small conductance channels) and chloride channels, (2) glutamate transporters such as EAAT1/GLAST and the L-cystine/glutamate antiporter. The role of H_2_S as an important mediator in cellular functions and physiological processes that are triggered by its interaction with ion channels/transporters in the eye will also be discussed.

## Introduction

The potential role of hydrogen sulfide (H_2_S) as a regulatory mediator has stimulated a surge of interest in its biological significance in cellular functions in the human body. This colorless gas, known for decades only as a toxic environmental pollutant has been found to be produced in substantial amounts in mammalian tissues. The endogenous production of H_2_S in mammalian tissues is dependent on the activity of two pyridoxal-5′-phosphate dependent-enzymes, cystathionine β-synthase (CBS; EC 4.2.1.22) and cystathionine γ-lyase (CSE; EC 4.4.1.1). Both CBS and CSE are enzymes of the trans-sulfuration pathway that inter-converts L-methionine and L-cysteine but can also use L-cysteine as an alternative substrate to form H_2_S (Stipanuk and Beck, 1982; Erickson et al., 1990; Swaroop et al., 1992). Recently, a newly identified enzyme, 3-mercaptopyruvate sulfurtransferase (3MST), has been reported to be involved in the production of H_2_S (Shibuya et al., 2009a,b). Current biomedical research suggest that H_2_S is an important gasotransmitter in mammals, and is involved in several physiological and pathophysiological processes as diverse as learning and memory, inflammation, and the regulation of blood pressure (Abe and Kimura, 1996; Lowicka and Beltowski, 2007). In the cardiovascular system, H_2_S has been shown to play a pivotal role in maintenance of vascular tone (Hosoki et al., 1997; Zhao et al., 2001; Cheng et al., 2004; Webb et al., 2008) whereas in the central nervous system (CNS) this gas was found to exert a neuroprotective role on neurons and exhibit neurotransmitter-like function in the modulation of synaptic activities (Zhao et al., 2001; Kimura, 2002; Kimura et al., 2005, 2006; Szabo, 2007; Qu et al., 2008; Webb et al., 2008). Many of the cellular effects of H_2_S in the vasculature and brain have been reported to be mediated by ion channels and transporters.

There is ample evidence that H_2_S targets different ion channels to modulate varied physiological functions. Extensive studies in the vasculature and CNS demonstrate that H_2_S interacts with ion channels such as ATP-sensitive potassium (K_ATP_) channels, calcium (Ca^2+^) and chloride (Cl^−^) channels to regulate vascular tone, and exert its neurotransmitter and neuroprotective-like properties (Kimura and Kimura, 2004; Tang et al., 2010). In addition, there is evidence that the neuromodulatory role of H_2_S in cellular functions and physiological processes are triggered by its interaction with several transporter systems. H_2_S has been reported to enhance the activity of transporters, thereby facilitating the release of antioxidants that are essential for neuronal protection against excitotoxic damage (Lu et al., 2008; Kulkarni et al., 2009; Kimura, 2011a,b). Furthemore, through its interaction with transporters, H_2_S plays an important role in maintaining the redox balance and thus serves both as a neuroprotectant and neuromodulator.

In contrast to the central nervous and cardiovascular systems, there are few reports of the involvement of H_2_S in the regulation of ion channels and transporters in the eye. Given the important modulatory effects of H_2_S on different ion channels and transporter systems in cellular functions and disease conditions in the central nervous and cardiovascular systems, there is a great need for studies centered on the potential role of H_2_S as a signaling molecule in ocular tissues. Indeed, we have evidence that H_2_S can induce pharmacological effects in mammalian ocular tissues, alter sympathetic and glutamatergic neurotransmission, and play a regulatory role in signal transduction processes in the eye (Monjok et al., 2008; Opere et al., 2009; Njie-Mbye et al., 2010; Ohia et al., 2010). The presence of CBS and CSE, the biosynthetic enzymes for H_2_S have also been reported in several ocular tissues, (Persa et al., 2006; Pong et al., 2007) indicating the existence of a functional trans-sulfuration pathway and a potential physiological role for H_2_S as a gaseous neuromodulator in the eye. Understanding the regulatory role of H_2_S in ion channel and transporter modulation in the eye is critical to our expanding knowledge of this gasotransmitter in ocular neuropathies. In this review article we will discuss the interaction of H_2_S with different types of ion channels and transporter systems found in the eye. Our attention will be particularly devoted on the role of H_2_S as a molecule able to trigger cell signaling in ocular tissues.

## Hydrogen sulfide and the eye

**Figure 1:**
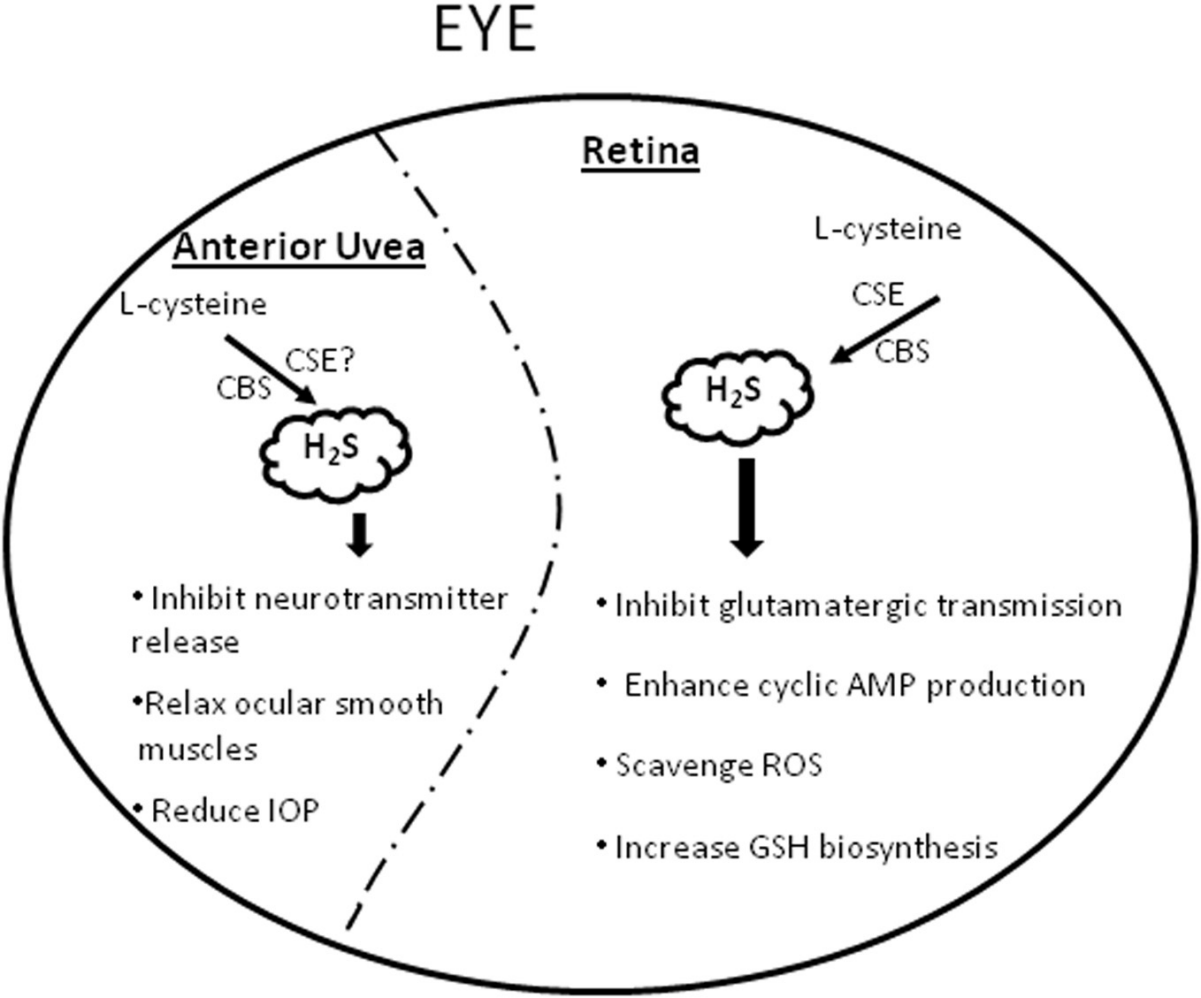
**A schematic representation summarizing the physiological and pharmacological effects of H_2_S in the eye.** GSH = glutathione, ROS = Reactive oxygen species, IOP = intraocular pressure.

Evidence from literature supports the presence of a functional trans-sulfuration pathway and a potential physiological/pharmacological relevance for H_2_S in the mammalian eye (De et al., 1974; Persa et al., 2006; Pong et al., 2007; Kulkarni et al., 2011). CBS and CSE, the primary enzymes of the trans-sulfuration pathway have been localized in mammalian ocular tissues (De et al., 1974; Persa et al., 2006; Pong et al., 2007). Moreover, deficiency of CBS has been linked to ocular disorders such as lens dislocation, retina degeneration, retinal detachment and acute glaucoma, (Kraus and Kozich, 2001) suggesting a physiological significance for this pathway in ocular tissues. Further support for a physiological relevance of H_2_S in mammalian ocular tissues was provided by us when we demonstrated the endogenous production of H_2_S in bovine ocular tissues (Kulkarni et al., 2011). Interestingly, the magnitude of H_2_S content corresponded to the reported expression of CBS and CSE enzymes in ocular tissues (De et al., 1974; Persa et al., 2006; Pong et al., 2007). In bovine retina, both CSE and CBS antagonists, propargyglycine (PAG), and aminooxyacetic acid (AOA) attenuated while the CBS stimulator, S-adenosyl-L-methionine (SAM) enhanced endogenous production of H_2_S, (Kulkarni et al., 2011) corroborating the involvement of these trans-sulfuration pathway enzymes in the production of H_2_S in retina. In addition to its *in situ* production, there is evidence supporting a pharmacological role for this gasotransmitter in mammalian ocular tissues (Figure 1). In the anterior uvea, we observed an inhibitory action of H_2_S (using sodium hydrosulfide, NaHS, and/or sodium sulfide, Na_2_S as donors) on both electrically evoked [^3^H]NE (norepinephrine) release and endogenous catecholamine concentrations in porcine iris-ciliary body in a concentration-dependent manner (Kulkarni et al., 2009). The inhibitory action of H_2_S donors on NE release was reversed by CBS and CSE antagonists, AOA and PAG respectively, suggesting that H_2_S attenuates sympathetic neurotransmission from isolated porcine anterior uvea by an effect that is partially dependent on its intramural biosynthesis. Moreover, H_2_S donors may exert their inhibitory action on sympathetic neurotransmission by a direct effect of this gasotransmitter on endogenous neurotransmitter release (Kulkarni et al., 2009). In another study, H_2_S donors exhibited an inhibitory action on carbachol-induced tone in isolated porcine irides that was dependent on endogenous production of prostanoids and the biosynthesis of H_2_S by CBS (Monjok et al., 2008). Whereas the nitric oxide (NO) synthase inhibitor, N (G)-nitro-L- arginine methyl ester (L-NAME) had no effect, the K_ATP_ channel inhibitor, glibenclamide (100 and 300 μM), blocked relaxations induced by NaHS, suggesting the involvement of K_ATP_ channels on the H_2_S on response in the anterior uvea (Monjok et al., 2008). In porcine irides, we observed an inhibitory action of L-cysteine (H_2_S substrate) that was dependent upon the endogenous production of H_2_S by CBS and CSE and was mediated by prostanoids and K_ATP_ channels (Ohia et al., 2010). Taken together, these data support a pharmacological role for H_2_S in the anterior uvea. So far, the potential therapeutic implications of the action of H_2_S in these tissues have not been fully elucidated. In preliminary studies, H_2_S donors reduced intraocular pressure (IOP) in normotensive rabbits (*Ohia et al., US Patent #8,092,838, Jan 10, 2012*). Similarly, the H_2_S-hybrid molecule ACS67 significantly reduced IOP in glaucomatous rabbits (Perrino et al., 2009) suggesting a potential application for H_2_S in the regulation of IOP. In spite of these findings, the exact role of the trans-sulfuration pathway in the anterior uvea and the mechanisms by which H_2_S regulates IOP remain unknown and merit further investigation.

In addition to the anterior uvea, pharmacological actions have been reported for H_2_S in mammalian retina as well (Figure 1). H_2_S donors inhibited amino acid neurotransmission from both isolated bovine and porcine retina by an effect that was dependent, at least in part, on intramural biosynthesis of H_2_S (Opere et al., 2009). Moreover, the gasotransmitter enhanced cyclic AMP production in bovine and porcine isolated neural retina and retinal pigment epithelial (RPE)-J cells by mechanisms that were dependent on biosynthesis of H_2_S by CBS and CSE and partially dependent on activation of the K_ATP_ channels (Njie-Mbye et al., 2010, 2012). Because an increase in retinal glutamate concentrations has been linked to retinal excitotoxicty, the ability of H_2_S to reduce glutamate release suggest a potential neuroprotective action in retinal neurons. Several investigators have since confirmed the neuroprotective effect of H_2_S in retina (Biermann et al., 2011; Mikami et al., 2011). Indeed, H_2_S donors protected mice retinal neurons from light-induced degeneration (Mikami et al., 2011). Similarly, H_2_S preconditioning conferred to protection of rat retina exposed to ischemia reperfusion injury (Biermann et al., 2011). The H_2_S-hybrid, ACS67 increased reduced glutathione levels, suggesting a potential neuroprotective role for this H_2_S-donor (Osborne et al., 2010). It is now apparent that H_2_S plays a dual role in biological tissues, being cytotoxic at higher and cytoprotective at lower concentrations of the gas (Martelli et al., 2010). The latter action, which has been demonstrated in various cell types and neurons, (Kimura et al., 2006; Sivarajah et al., 2006; Elrod et al., 2007) is partially ascribed to its ability to scavenge several reactive oxygen species (e.g., such as superoxide radical anion, hydrogen peroxide, peroxynitrie and hypochlorite) and increase GSH biosynthesis (Martelli et al., 2010). Several questions remain to be addressed, such as the role of molecular targets of H_2_S such as K_ATP_ channels in its neuroprotective action of H_2_S; integration of the trans-sulfuration pathway in retinal neurotransmitter pathways; interaction of H_2_S and transporters and other ion channels in the eye. Based upon the known pharmaocological role and protective mechanisms of H_2_S in biological systems, it is conceivable that H_2_S could find a significant application in ocular neuropathies, thereby opening up new molecular targets for management of ocular diseases.

## Regulation of ion channels by H_2_S in the eye

### Calcium channels (Ca^2+^) in ocular tissues

Calcium (Ca^2+^) is an essential ion that is involved in the regulation of several processes in the body such as signal transduction pathways, contraction, secretion, blood coagulation, gene expression, apoptosis, necrosis, cell division, and endocytocis (Williams, 1974; Shuttleworth, 1997; Berridge, 2005; Carafoli, 2005; Wimmers et al., 2007). Within the cell, free intracellular [Ca^2+^]_I_ content is tightly regulated at about 100 nM to maintain a steep inwardly directed concentration and electrochemical gradients across the cell membrane by an interplay of several Ca^2+^ channels, pumps, transporters, buffering systems and intracellular storage organelles (Bogeski et al., 2011). Several ion channels facilitate transmission of [Ca^2+^] ions across the membranes: the voltage-gated calcium channels (CaV), transient receptor potential (TRP) ion channels, transmitter-gated Ca^2+^ permeant ion channels and the store operated Ca^2+^ entry (SOCE) and Ca^2+^ released-activated Ca^2+^ (CRAC/Orai) channels (Bogeski et al., 2011). Although various potential molecular targets for calcium channels have been identified, only the L-type voltage-activated calcium channels have found wide therapeutic beneficial application. There is evidence in the eye for the existence of a calcium transport system. Ca^2+^ has been reported to play a key role in mammalian lens physiology and pathology. Excessive levels of Ca^2+^ have been implicated in cortical cataract and there is presence of Ca^2+^ linked receptors in the lens (Rhodes and Sanderson, 2009). Voltage-gated Ca^2+^ channels: transient (T-type) and dihydropyridine-sensitive long-lasting (L-type) channels, have been reported to be expressed in muller cells of the retina (Puro and Mano, 1991; Puro et al., 1996; Bringmann et al., 2000). The retinal pigment epithelium (RPE) has also been reported to expresses voltage- and ligand-gated Ca^2+^conducting channels (Wimmers et al., 2007). These channels act as regulators of secretory activity, and thus contribute to RPE function. Changes in Ca^2+^ channel function, or activity has been shown to lead to degenerative diseases of the retina (Wimmers et al., 2007).

### Effects of H_2_S on calcium channels (Ca^2+^) in ocular tissues

Despite the implication of Ca^2+^ in ocular physiology and pathology, there is a great need for studies centered on the regulatory role of H_2_S and its interaction with calcium channels. Only one study to date, has addressed the possible interaction of H_2_S and Ca^2+^ channels in the eye. In this study the authors' report that the production of H_2_S in retinal neurons is regulated by intracellular Ca^2+^, (Mikami et al., 2011) and in turn H_2_S can suppress Ca^2+^ channels by activating vacuolar type H^+^-ATPase (V-ATPase). Furthermore, the study also demonstrated that H_2_S can suppress the elevation of Ca^2+^ in photoreceptor cells by activating V-ATPase in horizontal cells and thus maintain Ca^2+^ homeostasis. From these observations, the authors conclude that H_2_S protects photoreceptor cells from the insult caused by excessive levels of light. Clearly results from this study provides a new insight into the regulation of H_2_S production and the modulatory interaction of H_2_S and Ca^2+^ channels in retinal transmission. In addition, the study postulates a cytoprotective effect of H_2_S on retinal neurons and provides a basis for the therapeutic target for retinal degeneration. Increasing knowledge about the properties of Ca^2+^channels in ocular tissuess especially the retina will not only provide a new understanding of ocular function but could also provide a better understanding of the role of H_2_S in ocular health and vision.

### Potassium (K^+^) channels in ocular tissues

Potassium ion (K^+^) channel family represents one of the most prominent and ubiquitous ion channels in living organisms where their physiological role range from regulation of the action potentials in excitable cells to regulation of transepithelial transport processes, intracellular pH, cell survival and growth factor secretion in non-excitable cells (Ashcroft and Gribble, 1999; Bauer et al., 1999; MacDonald and Wheeler, 2003; Warth, 2003; Masi et al., 2005). K^+^ channel family consists of four subfamilies, the inwardly rectifying K^+^ (K_ir_)-channels, voltage-gated K^+^ channels, Ca^2+^-activated K^+^ channels, and two-pore or leak K^+^-channels that are classified based upon number of transmembrane domains and electrophysiological properties. In the eye, K^+^ channels play central roles in maintaining ion, fluid balance and membrane potential. Several K^+^ channel subtypes such as voltage-gated K^+^ (Kv) channels and 4-aminopyridine (4-AP)-sensitive K^+^ channels are expressed in mammalian corneal epithelial cells (Rae, 1985; Rae et al., 1990; Rae and Farrugia, 1992). Studies have shown that changes in K^+^ channel activity modulate essential corneal epithelial functions needed for tissue homeostasis (Wolosin and Candia, 1987; Klyce and Wong, 1977). Furthermore, emerging evidence suggest that K^+^ channels play a crucial role in controlling apoptosis and proliferation in corneal epithelial cells (Lu et al., 2003; Roderick et al., 2003). Three major potassium currents (an outwardly rectifying current, an inwardly rectifying current, and a calcium-activated current) have been characterized in several mammalian lens epithelial cells (Rae, 1986; Cooper et al., 1991). These potassium conductances are essential for the maintenance of lens volume and transparency. Inwardly rectifying potassium (Kir) channel was reported to be highly expressed in bovine and human trabecular meshwork cells, (Llobet et al., 2001) as well as muller glial cells of the retina (Kofuji et al., 2002). K^+^ channels (Kv11; ether à-go-go related gene; erg) belonging to the family of voltage-gated K^+^ channels are present in mouse and human retina with the most abundant expression in rod bipolar cells. These channels are also found in the inner and outer plexiform layer, inner segments of photoreceptors, as well as the retina pigment epithelium (Cordeiro et al., 2011). These channels are vital for the control of the membrane potential in retinal neurons. Given the importance of K^+^ channel modulation in ocular tissues, evidence of an interaction between H_2_S and these channels in the eye is imperative for understanding the role of H_2_S as a signaling molecule in ocular functions.

### Effects of H_2_S on potassium (K^+^) channels in ocular tissues

The pharmacological effects of H_2_S in the vasculature and brain, has been reported to involve K^+^ channels. To the best of our knowledge there are no studies in the literature pertaining to the effects of H_2_S on K^+^ channels in the eye, except for those generated from our laboratory. In previous studies we have demonstrated that the pharmacological effects of H_2_S (using H_2_S–releasing compounds) in ocular tissues are partly mediated by K_ATP_ channels (Monjok et al., 2008; Kulkarni et al., 2009; Opere et al., 2009; Ohia et al., 2010). With the use of specific channel blockers, we report that H_2_S interacts with K_ATP_ channels to relax ocular smooth muscle, and alter sympathetic and glutamergic neurotransmission in the anterior uvea and retina (Monjok et al., 2008; Kulkarni et al., 2009; Opere et al., 2009; Ohia et al., 2010). Furthermore, we recently show that K_ATP_ channels are involved in the regulatory role of H_2_S in signal transduction processes in retina pigment epithelium cells (Njie-Mbye et al., 2012). It is reported that K^+^ channels play vital roles in cellular functions including vascular tone regulation, mediating neurotransmitter release, and neuroprotection in cardiovascular and CNSs (Yamada and Inagaki, 2005). Thus it is tempting to speculate a physiological role of H_2_S in ocular tissues that involves the activation of K^+^ channels.

### Chloride (Cl^−^) channels in ocular tissues

Chloride (Cl^−^) is one of the most prominent anions in the body that is involved in the regulation of a variety of important physiological and cellular functions such as volume homeostasis, organic solute transport, cell migration, cell proliferation, cell differentiation, and apoptosis. Unlike most physiological ions whose levels are tightly regulated within a limited range, the resting Cl^−^ ion concentration varies in different mammalian cell types and in developing cells (Wimmers et al., 2007). Cl^−^ conductance across membranes is facilitated by several pumps and co-transporters that are localized in plasma membranes and membranes of intracellular organelles. For example, chloride influx is facilitated by the Na^+^/K^+^/Cl^−^ co-transporters, Cl^−^/HCO_3_-exchangers, and Na^+^/-Cl^−^ co-transporters while efflux is achieved by the cell K^+^/-Cl^−^co-transporters and Na^+^-dependent Cl^−^/HCO_3_ – exchanger. Other channels and transporters expressed in intracellular membranes as well as Cl^−^ -binding proteins regulate intra-vesicular pH and Cl^−^ concentration (Duran et al., 2010). Several channels mediate passive flow of Cl^−^ ions across membranes. With exception of the transmitter-gated GABA and glycine receptors, these Cl^−^ channels are broadly classified into five subfamilies, the voltage-sensitive ClC subfamily, calcium-activated channels, high- (maxi) conductance channels, the cystic fibrosis transmembrane conductance regulator (CFTR) and volume-regulated channels (Verkman and Galietta, 2009). So far, only the voltage-sensitive ClC subfamily, CFTR and the transmitter-gated channels have been well described. In general, Cl^−^ channels are fairly non-selective for inorganic ions. Dysfunctional Cl^−^ channels have been linked to channelopathies such as myotonia congenita and cystic fibrocis (Duran et al., 2010). Cl^−^ channels are more abundantly expressed in the anterior segment of the eye due to Cl^−^ being the principal anion of aqueous humor secretion. Studies show that chloride efflux plays an important role in aqueous humor production and chloride channels present in the ocular ciliary epithelium are involved in aqueous humor homeostasis. Chloride currents have been reported to be present in bovine non-pigmented ciliary epithelium (NPE) and in transformed cultured human NPE (Chen and Sears, 1997). High-(maxi) conductance chloride channels are expressed in ciliary pigmented epithelial (PE) cells, (Do et al., 2004) whilst cAMP-activated Cl^−^ channels are present in the basolateral membrane of nonpigmented (NPE) ciliary epithelium (Edelman et al., 1995). CFTR is functionally expressed in corneal and conjunctival epithelium, corneal endothelium, and RPE (Shiue et al., 2002; Sun and Bonanno, 2002; Turner et al., 2002; Blaug et al., 2003; Levin and Verkman, 2005; Reigada and Mitchell, 2005). CFTR expression patterns in these tissues suggest the involvement of these chloride channels in regulation of tear film volume, corneal hydration and transparency, aqueous humor volume and IOP, and subretinal compartment size and ionic composition. In the retina, several Cl^−^ transporter and channels including the Na^+^/K^+^/Cl^−^ cotransporters, CFTR, and the voltage-sensitive ClC subfamily were reported to be highly expressed in the pigment epithelial layer (Zhang et al., 2011).

### Effect of H_2_S on chloride (Cl^−^) channels in ocular tissues

The activation of Cl^−^ channels by H_2_S in the CNS has been shown as a protective mechanism for neurons from oxytosis (Tang et al., 2010). Electrophysiological evidence demonstrates that H_2_S interacts with Cl- channels in the vasculature (Tang et al., 2010). To the best of our knowledge there are no studies reporting the interaction of H_2_S with Cl^−^ channels in the eye. The observation of the presence of chloride channels in ocular tissues especially in the anterior uvea, coupled with evidence of channel activation by H_2_S in non-ocular tissues, suggest possible regulation of chloride fluxes by H_2_S in the eye with neuroprotective consequences and IOP lowering effects.

## Regulation of transporters by H_2_S in the eye

### Excitatory amino acid transporter/glutamate aspartate transporter (EAAT/GLAST) in ocular tissues

Glutamate is the major excitatory neurotransmitter in the mammalian CNS. Under normal physiological conditions, extracellular glutamate is tightly regulated (resting levels ≤1 μM) by five distinct excitatory amino acid transporters, EAAT1 (glutamate/aspartate transporter [GLAST]); EAAT2 (glutamate carrier [GLT-1]), EAAT3 (excitatory amino acid carrier 1 [EAAC1]), EAAT4 and EAAT5 (Zerangue and Kavanaugh, 1996; Levy et al., 1998). Excessive extracellular glutamate is known to lead to excitotoxicty in neuronal tissues. Thus, EAAT transporters play the essential role of rapidly terminating synaptic transmission, maintaining low ambient extracellular glutamate while simultaneously conserving neuronal glutamate for reuse via the glutamate-glutamine cycling system (Copenhagen et al., 2002; Zou and Crews, 2005). Glutamate transporter uptake activity is accompanied by a net inward movement of positive ions (3Na^+^:1H^+^ co-transport versus K^+^ counter-transport) (Kanner, 2006) and could be coupled to Na, K-ATPase pump (Rose et al., 2009). Glutamate transporters exhibit differential distribution in different tissues. In the mammalian retina, using immunocytochemical studies, EAAT1 (GLAST) has been shown to be localized in Muller cells (Rauen et al., 1996; Pow and Barnett, 1999). EAAT2 have been identified in cone photoreceptor and bipolar cells (Rauen and Kanner, 1994) while EAAT3 (EAAC1) in inner retinal neurons (Rauen et al., 1996). EAAT4 is localized in retinal astrocytes (Ward et al., 2004) and EAAT5 in photoreceptors, bipolar cells and in some muller cells (Arriza et al., 1997; Pow and Barnett, 2000). Interestingly, EAAT5 is found exclusively in retina (Pow and Barnett, 2000). The sodium-dependent glutamate–aspartate transporter (GLAST or EAAT1) is the major glutamate transporter in muller cells. This glutamate transporter (EAAT1/GLAST) maintains extracellular glutamate at a low level to ensure a high signal-to-noise ratio for glutamatergic neurotransmission and thus shield neurons from excitotoxic damage. To the best of our knowledge there are no studies till date that have examined the effect of H_2_S on glutamate transporter in ocular tissues. Only one study, by our laboratory has demonstrated that H_2_S donors caused an attenuation of glutamatergic transmission in mammalian retinae (Opere et al., 2009). Although the exact mechanism of action is not clear,it is feasible that H_2_S donors could decrease glutamatergic transmission in mammalian retinae due to involvement of EAAT.

### Cystine/glutamate antiporter (system x^−^_C_) in ocular tissues

The cystine/glutamate antiporter (System x^−^_C_) is responsible for the Na^+^ independent electroneutral exchange of cystine and glutamate. It is a member of the heteromeric amino acid transporter family which is composed of a heavy subunit and a corresponding light subunit linked by a disulfide bridge (Lim and Donaldson, 2011). System x^−^_C_ is regulated by extra- and intracellular gradients of glutamate which drives the import of cystine coupled to export of glutamate (Fiorucci et al., 2006; Lowicka and Beltowski, 2007). System x^−^_C_ has two major functions. First, it mediates cellular uptake of cystine for the maintenance of intracellular levels of glutathione, essential for protection of cells from oxidative stress. Second, it is instrumental in maintaining the redox balance between extracellular cystine and cysteine (Lo et al., 2008). In the eye, system x^−^_C_ has been identified in veterbrate lens) (Lim et al., 2005) and different parts of the mamamlian retina including the retinal endothelial cells, outer plexiform of retina, muller cells, retinal pigment cells and retinal ganglion (Kato et al., 1993; Bridges et al., 2001; Hosoya et al., 2002; Tomi et al., 2002; Dun et al., 2006). Oxidative damage of proteins is believed to underlie several major eye diseases such as age related nuclear (ARN) cataract, age related macular degeneration (AMD) and diabetic retinopathy (Lo et al., 2008). GSH, a major and potent antioxidant in the cells may prevent or slow down the progression of such diseases by protecting the thiol groups of proteins and minimizing oxidation-induced protein aggregation formation. However, oxidative stress alters rate of conversion of cysteine to glutathione and leads to depletion of glutathione levels (Fiorucci et al., 2006). System x^−^_C_ plays an important role in maintaining elevated intracellular levels of glutathione and serves as a potential therapeutic target for a number of ocular diseases.

### Effects of H_2_S on cystine/glutamate antiporter (system x^−^_C_) in ocular tissues

In the CNS, H_2_S demonstrates cytoprotective effect by protecting neurons and astrocytes, major type of glial cells from oxidative stress (Kimura, 2011a,b). H_2_S enhances the activity of system x^−^_C_ and thus significantly increases the transport of cystine into neurons to increase the levels of substrate cysteine, for glutathione synthesis. Even in the presence of glutamate, H_2_S significantly reverses the inhibition of cystine transport by glutamate (Kimura and Kimura, 2004). Based on the conclusions from these studies (Kimura and Kimura, 2004; Kimura, 2011a,b) in CNS, it will be interesting to investigate the effect of H_2_S on system x^−^_C_ transporter in astrocytes and muller cells in retina under oxidative stress. Since, muller cells are the primary sites of glutathione localization in the retina, and the retina is extremely vulnerable to oxidative stress, understanding the function of H_2_S on system x^−^_C_ in muller cells could play a pivotal role in protecting the retina from a variety of retinal diseases, such as diabetic retinopathy, age-related macular degeneration, and glaucoma. In the eye, there is evidence from few studies that demonstrate an increase in GSH production, following application of H_2_S releasing drugs such as ACS67, ACS1. Although the mechanism of action is not clear, the authors suggest that intracellular cysteine levels are enhanced indirectly to form GSH by H_2_S stimulation of glutamate/cystine antiporters (Sparatore et al., 2009; Perrino et al., 2009; Osborne et al., 2010). Moreover, as mentioned above, a study by Opere and Ohia (1997) had demonstrated the inhibitory action of H_2_S donors on glutamatergic transmission in mammalian retinae. So, it is plausible that H_2_S donors can render there cytoprotective effects by upregulating system x^−^_C_ transporter in the retina and thereby increasing the production of glutathione. As the exact mechanism of action needs to be investigated, there is very demanding need to understand the potential role of H_2_S on the system x^−^_C_ transporter in the eye.

### Cysteine transporter in ocular tissues

Cysteine transporters are widely distributed in various cell types including the muller cells of retina. Cysteine transporters readily import cysteine into the cell for direct conversion to glutathione (Bringmann et al., 2009; Mathai et al., 2009; Kimura, 2010). So far only one study by Kimura (2010) on brain cortex has shown the effect of H_2_S on cysteine transporters. The study demonstrates that H_2_S (acting as a reducing agent) reduces cystine into cysteine in the extracellular space and makes cells efficiently transport cysteine into cells for GSH production. Moreover authors state that, the contribution of cysteine transport to the production of GSH is much greater than that of cystine transport. This production of GSH is further enhanced by H_2_S under conditions of oxidative stress caused by excessive glutamate toxicity. To the best of our knowledge there is a lack of studies on cysteine transporters in the eye and no evidence of the effect of H_2_S on these transporters in ocular tissues. Although the production of H_2_S in the eye is not well understood as there is only one study, from our laboratory showing that this gasotransmitter is endogenously produced in ocular tissues (Kulkarni et al., 2011), it is possible that the release of H_2_S may regulate the transport of cysteine in ocular tissues, thus facilitating the production of GSH. This increase in the levels of GSH by H_2_S may contribute to the potential protective effect of H_2_S.

## Concluding remarks

**Figure 2:**
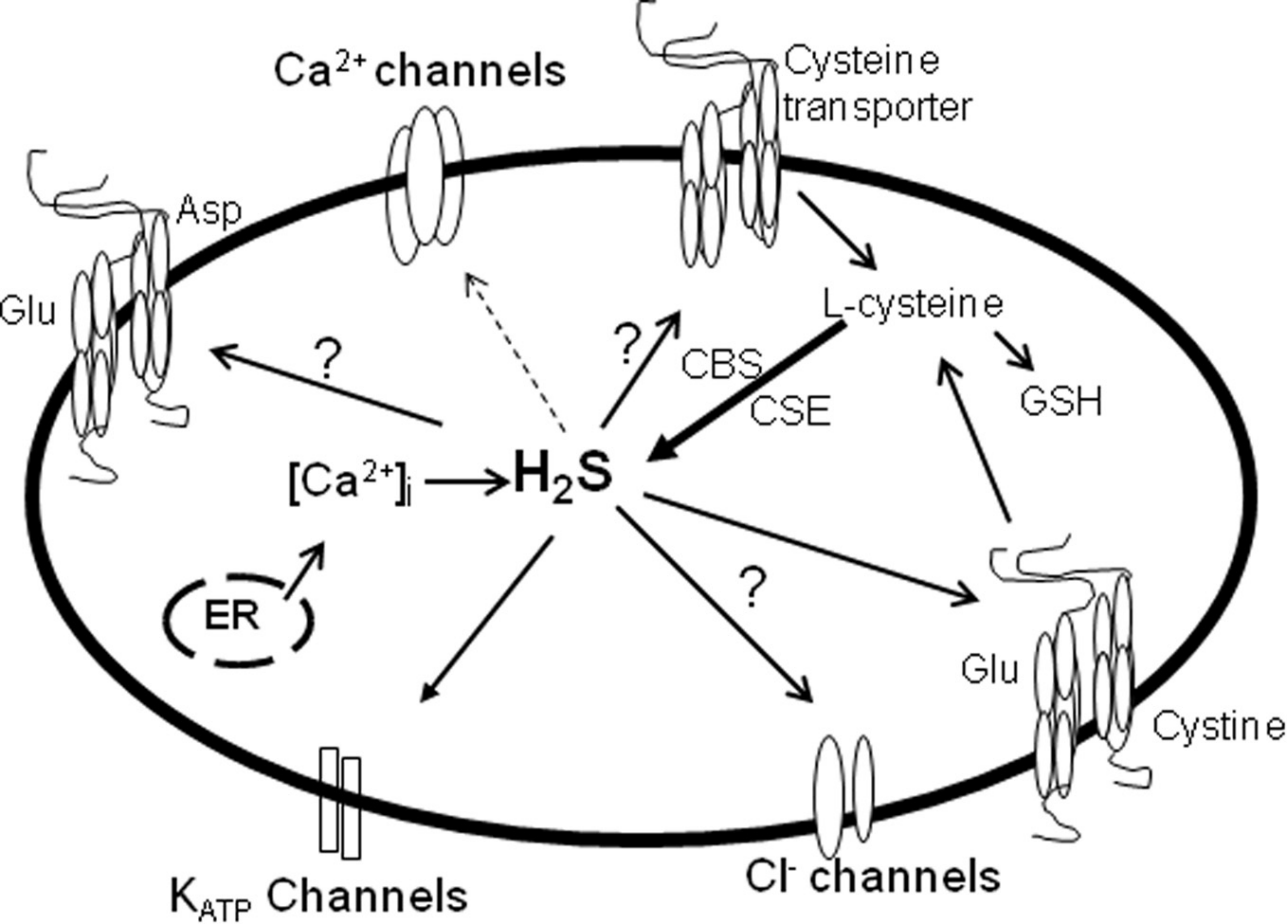
**The effects of H_2_S on ion channels and transporters in the eye.** Solid arrow, stimulatory; dotted arrow, inhibitory. Studies on the effects of ion channels and transporters in the eye and/or their interaction with H_2_S is lacking. H_2_S which is formed by CBS and CSE activities, stimulates K_ATP_ channels and the cystine/glutamate antiporter thereby regulating ocular smooth muscle relaxation, neurotransmitter release and oxidant/antioxidant balance (solid arrow). The production of H_2_S can be regulated by intracellular Ca^2+^, and in turn H_2_S can suppress Ca^2+^ channels to exert its neuronal effects (dotted arrow). Whether H_2_S activates or inhibits glutamate aspartate transporter, cysteine transporter and Cl^−^ channels remains to be determined.

Current evidence suggests that ion channels and transporters are present in ocular tissues and are involved in the regulation of vital cellular functions related to vision processes (Figure 2). However, the interaction of these signaling cascades with H_2_S in the eye is lacking. Clearly, there exists ample evidence that portrays the critical role H_2_S plays in physiological and pathophysiological processes in the human body. Furthermore, there is enough data that demonstrates that H_2_S targets different ion channels and transporters to modulate varied physiological functions in the central nervous and cardiovascular systems. Our current knowledge of such interactions in these systems should help facilitate research targeted on investigating the neuromodulatory role of H_2_S in the eye and its interaction with ion channels and transporters that are play pivotal roles in the preservation of vision. Indeed, future studies are warranted to examine the pharmacological effects of H_2_S on different types of ion channels and transporters in ocular tissues. Altered effects of H_2_S on ion channels and transporters, under different pathophysiological conditions in the eye also calls for intensive investigation.

### Conflict of interest statement

The authors declare that the research was conducted in the absence of any commercial or financial relationships that could be construed as a potential conflict of interest.
